# Digital Storytelling for Health-Related Outcomes in Older Adults: Systematic Review

**DOI:** 10.2196/28113

**Published:** 2022-01-12

**Authors:** Jennifer Stargatt, Sunil Bhar, Jahar Bhowmik, Abdullah Al Mahmud

**Affiliations:** 1 Department of Psychological Sciences School of Health Sciences Swinburne University of Technology Hawthorn Australia; 2 Department of Health Sciences and Biostatistics School of Health Sciences Swinburne University of Technology Hawthorn Australia; 3 Centre for Design Innovation School of Design Swinburne University of Technology Hawthorn Australia

**Keywords:** digital storytelling, mental health, aging, dementia, reminiscence, memory, systematic review, older adults

## Abstract

**Background:**

Older adults face a unique set of challenges and may experience a range of psychological comorbidities. Digital storytelling is an emerging tool for sharing and recording lived experiences and may have the potential to support well-being but is yet to be systematically reviewed for use among older adults.

**Objective:**

The aim of this review is to examine the methods for creating digital stories, the health-related outcomes associated with creating digital stories, and the potential for implementing digital storytelling with older adults.

**Methods:**

We systematically searched electronic databases to identify articles published in English that reported on at least one health-related outcome of digital storytelling for participants aged ≥60 years. Data were extracted and synthesized using qualitative content analysis and summarized in tables. The methodological quality of the studies was assessed using the Mixed Methods Appraisal Tool.

**Results:**

A total of 8 studies were included in the review. Participants were primarily community-dwelling older adults living with dementia, involving family caregivers and professional care staff. Studies have taken various approaches to digital storytelling and reported diverse benefits associated with digital storytelling, including improvements in mood, memory, social engagement, and quality of relationships. Although the potential for implementation was not widely examined, some studies have presented evidence for acceptability and feasibility. Generally, studies were of high quality, despite the absence of comparator groups and confounder analyses.

**Conclusions:**

The evidence reviewed suggests that despite the various approaches taken, digital storytelling shows promise as an effective approach for supporting well-being in older adults.

**Trial Registration:**

PROSPERO International Prospective Register of Systematic Reviews CRD42019145922; https://www.crd.york.ac.uk/prospero/display_record.php?ID=CRD42019145922

**International Registered Report Identifier (IRRID):**

RR2-10.2196/15512

## Introduction

### Background

The number of people aged ≥60 years is growing rapidly, worldwide [1]. Although most older adults experience positive mental health, a significant number of older adults experience psychological comorbidities, including depression, anxiety, and loneliness [2-4]. Furthermore, for those who require care, moving into a long-term care setting can elicit feelings of reduced personal autonomy, purpose, and sense of self, as familiar possessions and activities that support the person’s identity are often lost [5,6].

Autobiographical storytelling may improve psychological well-being in older adults living in community or long-term care settings. Recalling personal stories may encourage beliefs of self-mastery and problem solving, improve mood by eliciting pleasant memories, and support ego integrity—accepting and integrating one’s highs and lows and finding a meaning or greater purpose in life events [7,8]. Storytelling may enable older adults to feel recognized, affirmed, empowered, and accomplished and may assist in building resilience [8-10]. Reviews of studies have suggested that activities that include reminiscence about one’s life story may improve subjective psychological well-being, quality of life, mood, and cognition [11-14]. In addition, reviews have found that activities that involve reminiscence have the potential to improve quality of life, cognition, communication, and activities of daily living in older adults living with Alzheimer disease and dementia [15,16].

Tangible artifacts, such as books, collages, and memory boxes, are often created as a product of such life story work with older adults to record, retain, and share stories with others [17]. An integrative review found that life story work, in which an end product was created, assisted in maintaining a sense of identity and enhancing relationships for older adults in long-term care settings, most of whom were living with dementia [18]. For older people living with dementia, life story work can stimulate memories, enhance person-centered care, and promote conversations with family members and carers [17,19,20].

Owing to the advances in the capability and accessibility of multimedia technologies, it is now possible to produce digital story artifacts with relative ease. Digital storytelling is a process that involves using multimedia technology to combine images, sounds, and narration to create a film that documents one’s lived experiences [21]. It is an interdisciplinary approach used in educational settings [22,23], participatory research [24,25], and community engagement [26]. It can be facilitated in groups or one-on-one with individuals, with common aims to engage participants to record and share their lived experiences to educate others [27], enhance community engagement [28], and deepen their understanding of their personal stories [29]. For example, Lambert [21,30] engages people to create films about their own lived experiences, in which each story lasted for 3 to 5 minutes.

The use of digital storytelling, broadly defined, to improve the health of older adults is an emerging area of research. Similar to traditional life story artifacts, using digital technology to create and share autobiographical stories may enable older adults to benefit from the experience of being listened to and the opportunity to express their emotions and their identity [31]. To date, studies suggest that such digital storytelling is used with older adults, including those living with dementia, in a variety of ways—as a tool to improve mood [32,33], enhance memory [33], increase social connectedness [32,34,35], enhance the quality of care [34], and promote intergenerational relationships and learning [36-38].

With the increased accessibility of digital technologies, stories about past experiences can be easily documented in the form of narratives. However, the methods for creating such stories, the outcomes of such stories for personal well-being, and the potential to implement digital story activities within community or long-term care settings remain to be systematically reviewed. Given that digital storytelling could be beneficial for older adults, a systematic exploration of this growing body of literature is warranted.

### Objectives

This review aims to answer the following questions:

1. What health-related outcomes have been reported in relation to digital storytelling activities in older adults?
2. What methods for conducting digital storytelling activities for older adults have been reported?
3. What is the potential for implementation (eg, acceptability, appropriateness, and feasibility) of digital storytelling activities for older adults?

## Methods

### Registration

The peer-reviewed systematic review protocol [39] was developed following the PRISMA-P (Preferred Reporting Items for Systematic Reviews and Meta-Analyses Protocols) guidelines [40] and registered with PROSPERO (CRD42019145922). This systematic review adhered to the recommendations of the PRISMA (Preferred Reporting Items for Systematic Review and Meta-Analyses) guidelines [41].

### Eligibility Criteria

#### Study Designs

In this review, we included all possible study designs, including quantitative (eg, randomized, nonrandomized, quasi-randomized, and cluster-randomized controlled trials; pilot trials; open trials; case studies; cross-section studies; cohort studies; and case-control studies), qualitative, and mixed methods studies, provided that at least one health-related outcome was reported concerning digital storytelling. No study designs were excluded provided all other inclusion criteria were met. This decision to include a range of study designs was pragmatic, given that digital storytelling remains a relatively new area of research across various health care disciplines. Hence, the breadth of studies would provide an overview of the methods, outcomes, and implementation characteristics of digital storytelling with older adults.

#### Participants

We included studies in which all participants were older adults, defined for this review by the United Nations classification of those ≥60 years [1]. No exclusions were made based on participant health—studies were included regardless of dementia, mild cognitive impairment (MCI), and other illnesses. Studies were included regardless of participant setting (eg, community, long-term care, and hospitals).

#### Interventions

Digital storytelling was defined as creating a short (usually 3-5 minutes) multimedia clip (eg, images, videos, narration, and music) focused on the lived experience of older adults. We did not exclude studies based on story length or the level of participant involvement in production; digital stories may have been produced entirely by participants, produced on their behalf, or cocreated by the participants and others such as researchers, carers, or volunteers.

#### Comparator Groups

Studies were included regardless of whether they had a comparator group and irrespective of the type of comparator group included.

#### Outcomes

##### Health-Related Outcomes

Studies examining any outcome related to physical, psychological, or social health were included in the review. Examples of such outcomes include mood, memory, quality of life, and social engagement. Studies were included irrespective of whether these outcomes were measured quantitatively (eg, using validated psychometric assessment tools) or assessed qualitatively (eg, as a result of participant interviews, which were transcribed and analyzed thematically). Studies were excluded if digital storytelling was used in conjunction with another activity where the effects of digital storytelling alone were not reported or could not be ascertained.

##### Methods of Storytelling

The outcomes related to methods used in digital storytelling that were reviewed were as follows: (1) process characteristics (duration of participation and level of involvement in production) and (2) product characteristics (presence of audio-visual components such as still images, videos, music and narration, story theme, and length of the story).

##### Implementation Characteristics

We reviewed 8 implementation characteristics. We operationalized the implementation characteristics detailed by Peters et al [42] based on the framework by Proctor et al [43]. Implementation characteristics were acceptability, adoption, appropriateness, feasibility, fidelity, cost, coverage, and sustainability.

##### Report Characteristics

We included studies for which we could access the full-text reports, published in scholarly journals or unpublished in the case of dissertations and theses, written in English, and with no restrictions on country of origin or year of publication.

### Search Methods

An exhaustive search was conducted in October 2019. We searched the following databases using a planned strategy to identify published studies: MEDLINE (Scopus), Embase (Scopus), PubMed, PsycINFO, Web of Science, CINAHL (EBSCO), Academic Search Complete (EBSCO), Abstracts in Social Gerontology (EBSCO), Psychology and Behavioral Sciences Collection (EBSCO), Health Source: Nursing Academic Edition (EBSCO), and SocINDEX (EBSCO). Unpublished studies were searched using ProQuest Dissertations and Theses and Open Access Theses and Dissertations. We also conducted backward citation tracking to search the reference lists of all the included studies to identify any relevant studies that may have been missed.

The selected search terms were chosen to describe the characteristics of the population and the activities necessary for the review. An example search (Scopus) is as follows:

TITLE-ABS-KEY (older adult* OR elder* OR older person* OR older people* OR dementia) AND TITLE-ABS-KEY (story OR stories OR storytelling OR biographi* OR biography*) AND TITLE-ABS-KEY (digital OR multimedia OR virtual).

### Data Collection and Analysis

#### Overview

Titles and abstracts produced by the database searches were collated using reference management software and duplicates were removed. Titles and abstracts were screened to remove obviously irrelevant reports before full texts of potentially relevant studies were assessed for inclusion based on the eligibility criteria. Using a pilot-tested data collection form, data were extracted from the included studies and synthesized. If there were multiple reports of a single study, they were identified and the extracted data were presented as findings from a single study. A total of 2 reviewers were involved in the screening of all the abstracts and full-text records and in the data extraction process. Discrepancies were resolved through discussion and consensus. Where necessary, a third reviewer was included in the discussion and a decision was made based on group consensus.

The corresponding authors of the studies were contacted via email for information to (1) clarify study eligibility for the review, (2) clarify or provide additional data to assist with data extraction, and (3) clarify or provide additional information to assist with quality assessment. If the authors could not be contacted to clarify study eligibility, the study was excluded. In instances where the authors could not be contacted for data extraction or quality assessment purposes, studies were included with missing data. Of the 8 authors, 5 (63%) authors responded to emails from the reviewers.

Owing to the considerable heterogeneity of study designs and study types, a statistical meta-analysis was not feasible. Data were synthesized using a qualitative content analysis guided by the framework provided by Popay et al [44]. Study findings were synthesized using textual descriptions and tabulation. Qualitative studies were analyzed for themes by the first author (JS) [45].

#### Risk of Bias

The Mixed Methods Appraisal Tool (MMAT) was used to assess the methodological quality of the included studies [46]. The MMAT was chosen as it allowed for the appraisal of a variety of study designs, including quantitative nonrandomized, qualitative, and mixed methods studies. It comprised distinct sets of criteria to assess the validity of a study for each of the various study designs [47]. A total of 2 reviewers independently appraised all the studies and resolved the discrepancies through discussion. Where necessary, a third reviewer was included in the discussion and a decision was made based on group consensus. A critical discussion of the appraisal, both within and across studies, is presented.

## Results

### Study Identification

**Figure 1:**
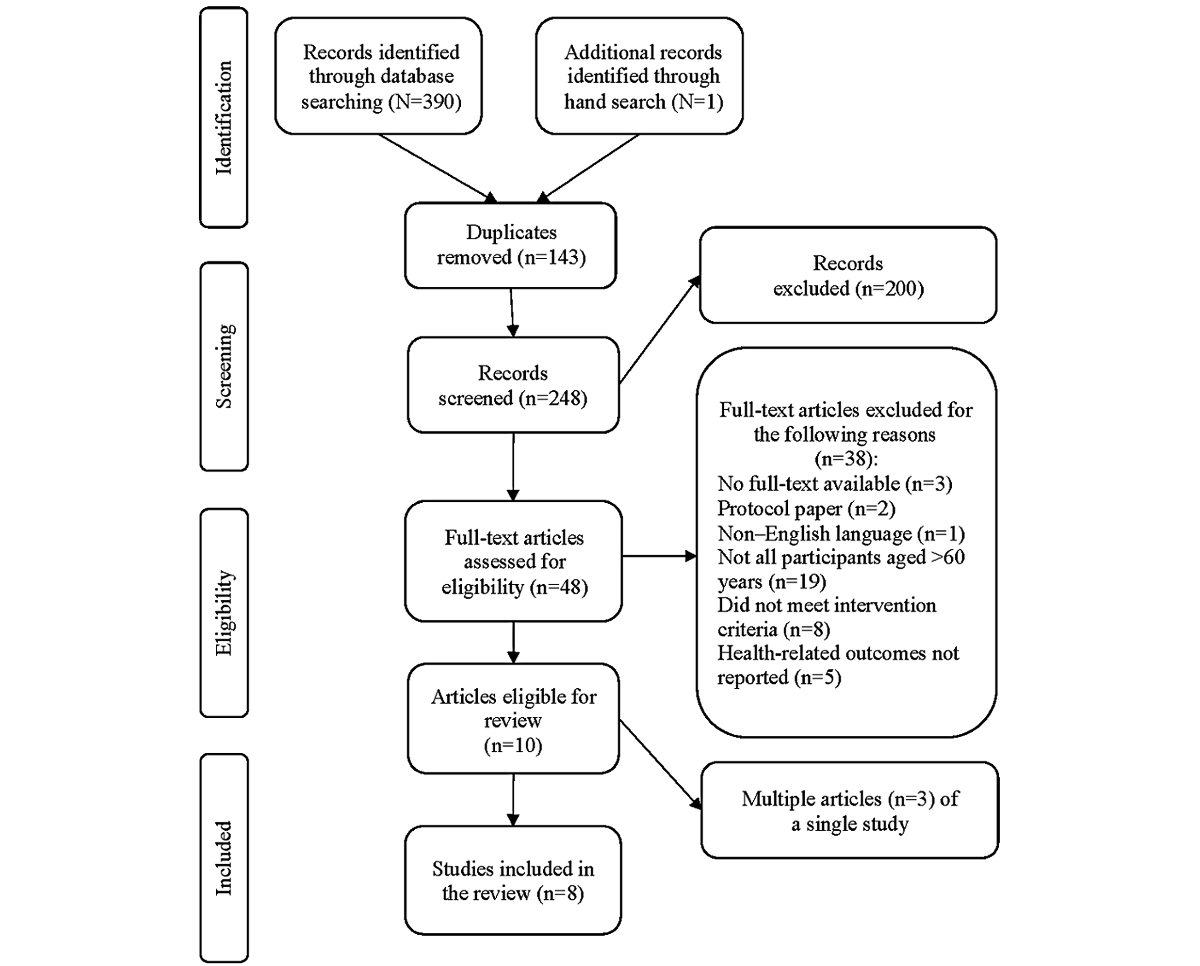
PRISMA (Preferred Reporting Items for Systematic Reviews and Meta-Analyses) flow diagram of study selection.

A PRISMA diagram of the selection process and flow of records at each stage is shown in Figure 1. Of the 391 records identified, duplicates were removed, and 248 (63.4%) records were screened for titles and abstracts. The full texts of 19.3% (48/248) of the records were reviewed for inclusion and exclusion criteria. Consensus was reached after independent review resulted in 90% agreement. Of the 10 records that met the eligibility criteria, 3 (30%) records were related to the same study. These 3 records were linked and presented as a single study.

Therefore, 8 studies were included in this review. Table 1 summarizes the study information, process characteristics, and product characteristics and Table 2 summarizes the key health-related outcomes of these studies. Table 3 presents the implementation characteristics.

**Table 1:** Characteristics of the included studies (N=8).

Study	Study design	Sample description						Creation and content of stories
		Study population	Age (years), mean (range)	Diagnosis	Facility- or community-dwelling	Country		
Capstick et al [48]	Quantitative nonrandomized	10 (n=8, 80% female and n=2, 20% male)	87 (76-99)	Dementia	Long-term care facility	United Kingdom	Stories cocreated by participants and researchers; 1 hour per week for 6 weeks; stories consisted of still images (personal and generic), participant narration, music, and sound effects; 3.5-11 minutes in length.	
Filoteo et al [49]	Quantitative nonrandomized	14	≥60	Dementia (mild to moderate)	Community-dwelling (recruited from an outpatient neuropsychological clinic)	United States	Stories created by family members, in a single day; stories consisted of still images (personal), family member voiceover, and music.	
Subramaniam and Woods [33]	Mixed methods	6 (n=4, 67% female and n=2. 33% male)	82.2 (73-89)	Dementia (mild to moderate)	Long-term care facility	United Kingdom	Stories cocreated by participants and researchers; 1-1.5 hours per week over 7-10 weeks (mean 8.3 weeks); stories consisted of still and moving images (personal and generic), text captions, participant and family member voiceover, and music; 12-27 minutes in length (mean 18 minutes).	
Crete-Nishihata et al [50], Damianakis et al [51], and Smith et al [32]	Qualitative	12 (n=7, 58% female and n=5, 42% male)	79.6 (60-95)	Alzheimer disease or MCI^a^ (early to midstage)	Long-term care facility (n=2, 17%) and community-dwelling (n=10, 83%)	Canada	Stories cocreated by participants, family members, and 2 RAs^b^; 4-10 sessions over 2-12 months (mean 5.6 months); stories consisted of still and moving images (personal and generic), participant and RA voiceover, and music; 15-70 minutes in length (mean 39.1 minutes).	
Critten and Kucirkova [52]	Qualitative	3 (n=1, 33% female and n=2, 67% male)	83.3 (72-94)	Dementia (mild to moderate)	Community-dwelling (recruited from a day center)	United Kingdom	Stories cocreated by participants and family members over 7 weeks; stories consisted of still images (personal and generic), text captions, and participant voiceover; user views story at their own pace—length varied.	
O’Philbin [53]	Qualitative	6 (n=1, 17% female and n=5. 83% male)	Mean unknown (70-85)	Dementia (mild to moderate)	Community-dwelling	United Kingdom	Stories cocreated by participants and family members; 1-2 hours per week over 6 weeks.	
Park et al [54]	Qualitative	7 (n=3, 43% female and n=4, 57% male)	74 (69-80)	Dementia (early stage)	Community-dwelling	Canada	Stories cocreated by participants and family members; 7 sessions of 2 hours over 6 weeks; stories consisted of still images (personal and generic), participant voiceover, and music; 3-8 minutes in length.	
Sehrawat et al [55]	Qualitative	4 (n=3, 75% female and n=1, 25% male)	Mean unknown (73-82)	Healthy	Community-dwelling	United States	Stories cocreated by participants and young people; 6 sessions over 6 weeks, including a full-day workshop for production; stories consisted of still and moving images and participant voiceover.	

^a^MCI: mild cognitive impairment.

^b^RA: research assistant.

**Table 2:** Key outcomes of included studies (N=8).

Study	Measures or tools used	Mood and affect	Memory	Quality of relationships	Social connectedness	Other health-related outcomes
Capstick et al [48]	BCC^a^ coding frame (DCM^b^), BWP^c^, and Arnstein Ladder of Citizen Participation	—^d^	—	—	Level of social citizenship increased by approximately 3 rungs.	Significant increase in positive well-being scores (*P*<.05) and significant decrease in negative indicators of well-being (*P*<.05) at midpoint. Well-being did not significantly decrease at 1 week after DS^e^; participants spent greater percentage of time engaged in reminiscence, conversation, and creative expression from before test to midpoint and after test.
Filoteo et al [49]	ET^f^, STAI^g^, HADS^h^, NQOL^i^, and CQ^j^	Statistically significant improvements on ET, STAI, HADS, and CQ from before test to after test (*P*<.05).	—	—	—	No statistically significant improvement on NQOL from before test to after test (*P*>.05).
Subramaniam and Woods [33]	QOL-AD^k^, AMI^l^, GDS^m^, QCPR^n^, and open-ended questionnaire	Improvement in scores on GDS at 4 weeks following the completion of DS.	Improvement in scores on AMI at 4 weeks following the completion of DS.	Improvement in scores on QCPR at 4 weeks following the completion of DS; participants, family members, and staff reported that the DS triggered memories and positive affect for the participant and enhanced interaction with family members and staff.	—	Improvement in scores on QOL-AD at 4 weeks following the completion of DS.
Crete-Nishihata et al [50], Damianakis et al [51], and Smith et al [32]	Semistructured interview and video recordings of screening sessions	Participants, family members, and staff reported emotional impacts of DS (eg, pleasure, sadness, and satisfaction); instances of positive emotion (n=291), negative emotion (n=6), and positive and negative emotion simultaneously (n=16).	Participants, family members, and staff reported that DS triggered long-term memories.	Participants, family members, and staff reported enhanced communication with family members and staff.	—	Participants, family members, and staff reported benefits for participants’ sense of self.
Critten and Kucirkova [52]	Interviews, field notes, and observations	Researchers reported the process was enjoyable for all participants and they experienced positive feelings of confidence, empowerment, and increased self-esteem.	—	—	—	—
O’Philbin [53]	Interviews	Participants and family members reported pride and enjoyment.	Participants and family members reported DS evoked memories.	—	—	—
Park et al [54]	Unstructured interviews, field notes, and audio recordings of sessions	Participants and family members reported enjoyment and a sense of accomplishment.	—	Researchers observed that participants were engaged in their relationships with their family members and the facilitator.	—	—
Sehrawat et al [55]	Open-ended questionnaire and unstructured interviews	—	—	—	Participants reported valued connections with young people and reported an increase in social connectedness and network size.	Participants found the process cathartic and therapeutic; however, they reported minimal to no change in physical and mental health.

^a^BCC: behavior category code.

^b^DCM: Dementia Care Mapping.

^c^BWP: Bradford Well-being Profile.

^d^Not addressed in the study.

^e^DS: digital story.

^f^ET: emotional thermometer.

^g^STAI: State-Trait Anxiety Inventory.

^h^HADS: Hospital Anxiety and Depression Scale.

^i^NQOL: Neuro–Quality of Life Depression Scale-modified.

^j^CQ: caregiver questionnaire.

^k^QOL-AD: Quality of Life in Alzheimer Disease scale.

^l^AMI: Autobiographical Memory Inventory.

^m^GDS: Geriatric Depression Scale.

^n^QCPR: Quality of the Caregiving Relationship Questionnaire.

**Table 3:** Implementation outcomes (N=8).

Study	Acceptability^a^	Adoption^b^	Appropriateness^c^	Feasibility^d^	Fidelity^e^	Cost^f^	Coverage^g^	Sustainability^h^
Capstick et al [48]	A participant became upset after watching her DS^i^ that contained photos of a relative who had died.	—^j^	—	100% retention rate.	—	Use of free software (eg, Photo Story and Audacity).	—	The authors state that their step-by-step guide to participatory video is made available for others to replicate their work.
Filoteo et al [49]	—	—	—	—	—	“Low-cost” platform on a custom tablet for use on currently owned devices.	—	—
Subramaniam and Woods [33]	Reported no negative side effects. Enjoyed by all. Well received by relatives and staff. Disagreements with relatives regarding content and format were rarely encountered.	—	A total of 4 participants needed assistance to operate the DVD player; however, most reported preferring the digital form of their story over the previously made books. All participants needed someone to remind them to play the movie.	100% recruitment rate and 100% retention rate.	—	Used free software for production.	—	Widespread implementation requires consideration of time and skills—without the researcher, staff would have to take on the task of production.
Crete-Nishihata et al [50], Damianakis et al [51], and Smith et al [32]	There were varied viewing experiences, for example, after several viewings, 1 participant worried about how she could have made it differently and suggested that it should be editable. Strong rapport must be built among biographers, family members, and participants to resolve disagreements. Acknowledged that there are multiple interpretations of a life story and decisions must be made about including emotionally sensitive content. Personal media content is crucial.	—	Purposefully chose familiar technologies to enable easy adoption and integration (eg, television and DVD player). Still, some participants had trouble in operating the DVD player. Recognized that dementia severity may impact production participation.	52% recruitment rate (remaining participants declined owing to personal reasons) and 86% retention rate (1 dropout owing to death and 1 owing to time constraints).	—	Researchers worked for an average of 131.7 hours. As they became familiar with the process, they needed 60-90 hours to produce the DS. Family caregivers may not have time to do this without researchers. Production value varied, ranging from consumer-level to professional equipment. Inexpensive software was used.	—	A guide for families that may be interested in making their own DS is available.
Critten and Kucirkova [52]	Enjoyable for all participants who were all personally involved. The researcher– or carer–participant dynamic may influence the outcome of the study. Some may find this process difficult owing to sad memories.	—	Digital competence is necessary for participation as intended.	27% recruitment rate (remaining participants declined owing to technological limitations and time constraints) and 100% retention rate.	—	Used iPads and free software (Our Story).	—	—
O’Philbin [53]	For all participants, it was a mostly enjoyable experience. Some frustration was reported at not being able to recall specific things.	—	Some participants expressed “it’s not for everyone” (life story work). The digital nature of the program was a barrier for some participants.	50% recruitment rate and 86% retention rate (1 dropout owing to declining to be interviewed).	—	—	—	The Book of You service does not check and encourage implementation with previous users, but the author suggests this could be considered.
Park et al [54]	Although participants were not able to explicitly address how they felt or specify what they enjoyed, there was a level of participation and enthusiasm that indicated interest.	—	The existing protocol for the workshop was modified in this study for people with dementia by having shorter and condensed sessions with a smaller participant group. A total of 2 participants did not have computers and were unable to use the program without assistance. None were able to use the program independently. A participant could not read her story aloud owing to visual impairment.	88% retention rate (1 dropout owing to time constraints).	—	Used freely available video software (WeVideo). Participants needed support from the facilitator and relatives to use the technology.	—	—
Sehrawat et al [55]	All participants enjoyed the process. Some negative emotions were produced—necessary to consider including a debrief in future and allowing more time for participants to share their stories in the group activity.	—	Workshop day was very long—participants began to tire and lose focus.	100% recruitment rate and 100% retention rate.	—	Funding was required to pay the student research assistants. Used free video software (WeVideo).	—	—

^a^Reported agreeableness or enjoyment by or on behalf of participants.

^b^The intention, initial decision, or action to try to use the activity.

^c^The perceived fit for the target group, reported by or on behalf of the target group.

^d^Reported rates of recruitment and retention.

^e^Whether the activity was implemented as it was designed to be.

^f^Costs associated with the implementation of activity (eg, financial costs, time, and human resource).

^g^The degree to which the population eligible to benefit from the activity actually receive it.

^h^Whether the activity was reported to be maintained in the given setting.

^i^DS: digital story.

^j^Not addressed in the study.

### Study Characteristics

A total of 8 studies were reported in peer-reviewed journal articles (5/8, 63%), conference papers (2/8, 25%), and doctoral theses (1/8, 13%). Studies used qualitative (5/8, 63%), quantitative (2/8, 25%), and mixed methods (1/8, 13%) designs. The studies were based in the United Kingdom (4/8, 50%), the United States (2/8, 25%), and Canada (2/8, 25%). The quantitative and mixed methods studies used single-arm repeated measures (before-and-after) designs (3/8, 38%), of which 67% (2/3) of the studies used inferential statistics to test for statistical significance of pre–post differences in outcomes and 33% (1/3) of the studies showed descriptive statistics only. Qualitative studies used semistructured and unstructured interviews (6/8, 75%), of which 33% (2/6) also used field and observational notes. Qualitative analyses in these 6 studies were reported as content analysis (3/6, 50%) and thematic analysis (3/6, 50%). In 6 (75%) of the 8 studies, data on outcomes for participants and implementation characteristics were provided by participants and informants.

Quality assessment using the MMAT [46] indicated that study quality was acceptable overall and high for qualitative studies. Rating appraisal resulted in 75% agreement by 2 independent reviewers and consensus was reached via discussion. Studies were assessed on 5 quality criteria, with different sets of criteria for the qualitative, quantitative, and mixed methods studies. Of the 8 included studies, the 5 (63%) qualitative studies met all the MMAT criteria (5/5, 100%). For these studies, the qualitative approach was appropriate, using adequate data collection methods and presenting coherent findings that appeared to be adequately derived from and substantiated by data. The 25% (2/8) quantitative studies [48,49] did not account for confounders in their study design and analyses. In a third study, participants were identified as not representative of the target population. These studies were associated with MMAT scores of 3 and 4 out of 5. The mixed methods study did not explicitly produce an adequate rationale for using a mixed methods design and the quantitative component was assessed as not adhering to the quality criteria of the quantitative method, thus resulting in an MMAT score of 3 out of 5.

### Participant Characteristics

The 8 studies comprised 62 participants, with study sample sizes ranging from 3 to 14 (mean 7.75, SD 3.88). Of the 88% (7/8) studies that provided information regarding participant gender, there were 27 female participants and 21 male participants. Age was reported inconsistently across the studies. Across 63% (5/8) studies that reported statistics on age (38/62, 63%), participants were aged between 60 and 99 years (mean 81 years). Cognitive status was not reported for participants in 13% (1/8) studies (4/62, 6%)*.* Across the remaining 88% (7/8) studies, 52 participants were living with dementia, whereas 6 experienced MCI. In 63% (5/8) of the studies, dementia status was self-identified (32/62, 52%). Participants of 25% (2/8) of the studies (20/62, 32%) were assessed by the researchers for dementia status using the Diagnostic and Statistical Manual of Mental Disorders-IV or Diagnostic and Statistical Manual of Mental Disorders-V. Participants lived in long-term care facilities (18/62, 29%) or in the community (44/62, 71%). Community-dwelling participants were recruited from an outpatient neuropsychology clinic (14/44, 32%), local aging societies (11/44, 25%), day centers (9/44, 20%), or referred by health care professionals (2/44, 5%).

### Methods of Digital Storytelling

#### Process Characteristics

##### Duration

The time taken to produce digital stories varied across studies. In 6 (75%) of the 8 studies, digital storytelling was conducted for 6-10 weeks. In 13% (1/8) of studies, stories were in production for up to 52 weeks [51]. In another study, digital stories were produced in 1 day [49].

##### Frequency

In 88% (7/8) studies, production sessions were generally held weekly. In 13% (1/8) of studies, participants produced their digital stories without structured sessions [52].

##### Length

Digital stories were produced in sessions lasting for 1-2 hours.

##### Producers

In 13% (1/8) of studies, the digital story was created by a family member without involving the older adult [49]. In 38% (3/8) studies, digital stories were cocreated by the older adults and the researchers. In 50% (4/8) studies, digital stories were cocreated by older adults, researchers, and family members.

#### Product Characteristics

##### Audio-Visual Composition

Across the 88% (7/8) studies that provided information regarding product composition, all digital stories included images that were both personal and generic, that is, stock photos sourced from the internet. In 86% (6/8) of these studies, a voiceover was also provided by participants. Of these 6 studies, 2 (33%) studies also included voiceover by family members and 1 (13%) included voiceover by research assistants. In 13% (1/8) of studies, stories were narrated by a family member only. Among the 8 included studies, music was included in 5 (63%) studies, moving images in 3 (38%) studies, and sound effects in 1 (13%) study.

##### Themes

Although all stories focused on participants’ personal memories, 50% (4/8) of the studies identified specific themes of their participants’ stories. Of the 8 studies, 1 (13%) study focused on significant places and events from the ages of 5-30 years [48] and another study (13%) focused on the areas of family and work [52]. In 25% (2/8) of the studies, digital stories explicitly took a broader focus and presented chronological accounts of life events, for example, childhood, teenage years, and career. [33,51].

##### Length

Stories differed in length; across the 50% (4/8) studies that indicated length, digital stories ranged from 3 minutes to 70 minutes.

### Health-Related Outcomes

Health-related outcomes were explored across the included studies and clustered into five categories: mood and affect, memory, quality of relationships, social connectedness, and other health-related outcomes.

#### Mood and Affect

All 8 (100%) studies reported benefits related to mood and affect caused by digital storytelling activities.

Quantitative improvements in mood were reported in 25% (2/8) studies. Filoteo et al [49] reported that participants experienced significant (*P*<.05) pre–post improvements in anxiety, depression, overall emotional distress, and emotional functioning (as rated by family caregivers) after viewing their digital story. Subramaniam and Woods [33] found that participants reported a mean improvement in scores on standardized measures of depression (mean difference 1.84); however, such a difference was not evaluated for statistical significance.

Qualitatively, authors reported that digital storytelling fostered enjoyment [33,51-55] and other positive feelings including a sense of confidence [52], accomplishment, empowerment, self-esteem [52,54], enthusiasm [54], pleasure and satisfaction [51,55], and pride [53,54]. Some participants expressed grief and sorrow, but in the context of digital storytelling, this was considered in 13 (1/8) of studies as “natural expressions of loss, mitigated by the overall narrative of the life story” [33]. Damianakis et al [51] observed instances in which sadness and happiness were observed simultaneously.

#### Memory

Benefits associated with participant memory were reported across 88% (7/8) studies. Subramaniam and Woods [33] reported that participants experienced a mean improvement in scores on a standardized quantitative measure of autobiographical memory for factual knowledge (mean difference 8.92), providing some evidence for an effect of digital storytelling above and beyond the effect of the traditional life storybooks, which were created with participants before the digital storytelling activity. In contrast, autobiographical memory for specific events and incidents was overall highest following the traditional life storybook activity.

For participants across all 6 qualitative studies, digital storytelling provided a platform for stimulating long-term memories that may have been previously forgotten. Memories were elicited in various ways, including verbal prompts related to specific themes, for example, marriage and travel [51] or photographic material sourced from the internet [52]. Some participants and their family members identified that the digital story would serve as a valuable memory aid when their dementia progressed further [51,53].

#### Quality of Relationships

Across 63% (5/8) studies, digital storytelling activities improved participant relationships with their family members and professional caregivers.

The quality of caregiving relationships was assessed by Subramaniam and Woods [33], who reported mean improvements in scores on all subscales of a standardized quantitative measure (mean differences 0.83-6.83); however, such differences were not evaluated for statistical significance.

Qualitatively, 50% (4/8) studies described improved relationships between participants and family caregivers and professional care staff during or after digital storytelling [33,51,53,54]. Family members interviewed by Damianakis et al [51] reported that digital storytelling facilitated enhanced communication with their relative living with dementia, in both the quality and quantity of their interactions, as past events were remembered and discussed. Professional carers who were interviewed in 13% (1/8) of studies acknowledged that viewing digital stories would help them better care for people living with dementia, as it enabled a deeper appreciation of their unique histories and provided relevant talking points [33].

#### Social Connectedness

Of the 8 included studies, 4 (50%) studies addressed the extent to which digital storytelling enhanced social connectedness. All the 4 (100%) studies reported that digital storytelling improved interactions among the participants, with others involved in cocreating stories or with viewers of the stories.

In 3 (75%) of these 4 qualitative studies, involvement in digital storytelling provided opportunities for increased social engagement [53-55]. Sehrawat et al [55] reported that older adults formed meaningful intergenerational connections with the students they were paired with over the 6-week activity by connecting through the shared experience of storytelling. Park et al [54] observed enhanced relationships between the participants and their family caregivers. Family caregivers of community-dwelling people living with dementia who attended a 6-week digital storytelling group workshop spoke of the social benefits associated with their relative meeting others [53].

In 2 (50%) of these 4 studies, broader social connections were examined. Participants involved in the intergenerational activity described sharing their digital stories beyond the activity with their friends and family, producing what the authors refer to as a *wave of connectedness* [55]. In providing evidence for participants’ increased social citizenship, Capstick et al [48] reported that the digital stories of some participants were shared with the wider community (eg, on local history websites). Using a subjective measure of community engagement, the authors concluded that participants’ potential for social citizenship improved owing to their engagement in digital storytelling. Furthermore, the authors presented a case study example of a participant who was taken out to the local theater to watch a play about cycling after the staff at the facility viewed her digital story, which was focused on her early passion for cycling to cope with her challenging experience growing up in a care facility.

#### Other Health Outcomes

The authors also reported that digital storytelling was associated with improvement in general well-being, quality of life, and sense of self and identity. Such stories were also seen to provide older adults with opportunities for leaving a legacy.

Quantitative improvements in well-being were reported by Capstick et al [48]. The authors reported a statistically significant improvement in well-being at the midpoint of the 6-week activity period (*P*<.05) and no significant decrease in well-being at 1 week following the activity (*P*>.05). On an observational measure, participants spent a greater percentage of time engaged in reminiscence, conversation, and creative expression at the midpoint compared with baseline. This pattern was maintained at 1 week following the end of the activity.

Filoteo et al [49] administered a standardized quantitative measure of quality of life and found no significant improvement following digital storytelling (*P*>.05). Subramaniam and Woods [29] reported a mean improvement in scores on a standardized quantitative measure of quality of life in Alzheimer disease; however, this was not tested for statistical significance.

A total of 5 studies proposed that the digital storytelling process served to elicit and validate a sense of self and identity, evident throughout production, for example, in selecting desired images and music to best represent their story [51], in shared viewing of their stories, [48], and simply in having their stories recorded in a tangible fashion [52]. Relatedly, people living with dementia and their family members also noted the value of the opportunity to leave a personal legacy [33,51,54].

### Implementation Outcomes

#### Feasibility

Of the 8 included studies, 7 (88%) studies reported rates of recruitment or retention, indicating the potential for the feasibility of the digital storytelling activity. A small proportion of participants declined to be involved or dropped out of the studies owing to time constraints, death, difficulty in using the required technology, or other personal reasons (see Table 3 for the data).

#### Acceptability

All (8/8, 100%) studies reported that digital stories were agreeable and enjoyable. However, negative emotional reactions were noted in several studies, including some participants becoming upset during the activity, as the activity revived difficult memories and feelings of grief and loss [48,51,52,55]. Participants also became frustrated as they could not recall specific memories [53] and worried about how they could have made the story differently [51]. Notably, these instances of negative emotion were recorded as rare, occurring in only a small portion of participants per study.

#### Appropriateness

Of the 8 included studies, 6 (75%) studies discussed the appropriateness or the perceived fit of their activities. The primary consideration related to appropriateness was digital competence—authors noted that participants in some cases had difficulty in operating the required technology independently, affecting the production phase or their ability to view their digital story after the completion of the activity [33,51,52,54]. Of the 6 studies, 1 (17%) study cited that their protocol demanded too much attention and cognitive stamina of participants [55]. Some studies discussed the impact of dementia severity on the individual’s capacity to participate as intended and noted the need to adopt a flexible approach [51,54].

#### Cost

Cost was typically referred to in the context of equipment needs. In most instances, the authors reported using inexpensive or freely available photo and video software to produce digital stories using devices already owned by researchers or participants. Of the 8 studies, 1 (13%) study reported that funding was required to pay their student research assistants [55]. Only 13% (1/8) of studies provided an examination of the costs associated with time—researchers worked between 60 hours and 90 hours to produce lengthy digital stories, ranging from consumer-level to professional quality—with the authors acknowledging that family members may not have the time to undertake this activity themselves [51].

#### Fidelity

Studies did not assess whether the activity implement was as intended; fidelity of the protocols was not measured. Of the 8 included studies, 1 (13%) study stated that the authors’ existing protocol for the digital storytelling workshop was modified for the current sample (people living with dementia) before commencement of the activity [54]; however, even in this study, fidelity of the modified protocol was not assessed.

#### Adoption, Coverage, and Sustainability

The authors did not comment on adopting the activity in routine practice at the individual or organizational level or the coverage of the activity. Similarly, the authors did not report whether digital storytelling activities were sustained or maintained in their respective settings. However, the authors of 25% (2/8) of studies reported that they had prepared digital storytelling guidebooks that were available upon request for those interested in adopting their approaches [48,51].

## Discussion

### Principal Findings

The purpose of this systematic review was to explore the characteristics, outcomes, and potential for implementation of digital storytelling with older adults. This review summarizes the methods used to produce digital stories, features of the digital story products, health-related outcomes of digital storytelling, and implementational considerations of digital storytelling activities. The 8 studies that were reviewed comprised 62 participants aged between 60 and 99 years, most of whom had a diagnosis of dementia or MCI and lived in the community.

The review adopted an overly inclusive definition of digital storytelling, whereby restrictions were not placed on the level of participant involvement in creating stories, time taken to produce stories, or the length of the stories themselves, provided they satisfied all other inclusion criteria. Studies used markedly different methods for producing digital stories; in most studies, digital stories were cocreated by older adults and researchers and family members over several weeks. Some were short (eg, 3-5 minutes), consistent with Lambert’s protocols [21] and others were significantly longer, such as the *multimedia biographies* that were up to 70 minutes in length [32]. Digital stories were centered on the lived experiences of older adults, including stories of family, work, travel, and significant places and events. All digital stories used personal and generic images and voiceover narration, with many including music and some including moving images or video and sound effects.

Digital storytelling was associated with four overarching health-related outcomes: positive mood and affect; improved memory; enhanced relationships among older adults, family, and professional caregivers; and improved social connectedness. Approaches to assessing outcomes were heterogeneous, with outcomes assessed using a variety of quantitative and qualitative methods.

The reported potential for implementation varied across the studies. The included studies did not explicitly aim to examine the potential for the implementation of their activities. Using the implementation framework presented by Peters et al [42], the review found some evidence for the acceptability of digital storytelling through overall participant agreeableness and enjoyment. However, some authors reported noteworthy issues, including unanticipated negative emotions in few participants. Studies delivered activities that were considered appropriate for the target population; however, some issues related to fit were reported, including participants’ poor digital literacy and cognitive and emotional demands. There was evidence for feasibility as retention was relatively high and recruitment rates were adequate. Activities were delivered with a low financial cost; however, in some instances, the time commitment required for researchers, and research assistants, and family members was considerable. Studies did not discuss the adoption of the activity at the individual or organizational level, sustainability of the activity, or its coverage. Overall, findings from this review suggest that digital storytelling is implementable when activities are designed with careful consideration of the physical, cognitive, and emotional needs of the target population.

### Limitations

The MMAT [46] demonstrated that the included studies were generally of high quality.

However, several important questions remain unanswered. The single-group repeated measure designs of the quantitative studies pose a low level of evidence [56]. As none of the included studies used a comparator group, conclusions regarding the efficacy of digital storytelling compared with other or no activity cannot be made with confidence. Notably, the studies also did not conduct follow-up assessments to explore whether the effects were sustained for greater than a week [47] following digital storytelling. Studies did not generally aim to explore or account for confounding factors in their analyses—it remains largely unknown what components of the digital storytelling process, such as social interaction, stimulating and sharing of memories, feeling heard and valued, and producing a tangible digital story, have the most effect on outcomes. In addition, it remains unknown whether the outcomes of the digital storytelling process (after creation) are distinct from those related to digital story viewing (after viewing). Although some studies assessed outcomes at several time points, this question was not explicitly addressed and remains to be explored further. Nearly all participants (58/62, 93%) in the pool of reviewed studies were living with dementia or MCI. Hence, findings from this review may not be generalizable to cognitively healthy older adults.

Owing to the considerable heterogeneity of the studies, a qualitative content analysis was conducted to synthesize the evidence presented. The overly inclusive definition of digital storytelling was necessary to capture all relevant studies in this emerging literature; however, the robustness of the synthesis is moderately limited by the markedly differing purposes of digital storytelling and heterogeneous outcome measurement across only a few studies. A larger number of homogenous studies would allow a more confident account of the outcomes of digital storytelling.

Although an exhaustive search method was used, a librarian with expertise in search strategies was not consulted and forward citation tracking was not conducted. Gray literature, besides unpublished theses and conference papers, was not searched.

Furthermore, the primary focus of this review and the search criteria was to explore the health-related outcomes of digital storytelling activities—studies were only included if they reported at least one health-related outcome. Hence, studies were not included in this review if they did not report health outcomes; studies that only reported on methods or implementation potential of digital storytelling activities were not included in the current review.

### Conclusions

This is the first review to systematically survey the current state of digital storytelling literature for older adults. Despite varied approaches, the review found that, when used with older adults, digital storytelling is largely acceptable and feasible and shows potential for benefits related to mood and affect, memory, quality of relationships, and social engagement.

## Abbreviations

MCI: mild cognitive impairment
MMAT: Mixed Methods Appraisal Tool
PRISMA: Preferred Reporting Items for Systematic Review and Meta-Analyses
PRISMA-P: Preferred Reporting Items for Systematic Reviews and Meta-Analyses Protocols
